# Serum concentration and vascular expression of adiponectin are differentially associated with the diabetic calcifying peripheral arteriopathy

**DOI:** 10.1186/s13098-019-0429-7

**Published:** 2019-04-29

**Authors:** Carole E. Aubert, Sophie Liabeuf, Chloé Amouyal, Salim Kemel, Frédérique Lajat-Kiss, Jean-Marc Lacorte, Marine Halbron, Aurélie Carlier, Joe-Elie Salem, Christian Funck-Brentano, Ljubica Perisic Matic, Anna Witasp, Peter Stenvinkel, Franck Phan, Ziad A. Massy, Agnès Hartemann, Olivier Bourron

**Affiliations:** 1 Sorbonne Université, UPMC Univ, Paris 06, France; 2 Assistance Publique-Hôpitaux de Paris (APHP), Diabetology Department, Pitié Salpêtrière Hospital, 47-83 Boulevard de l’Hôpital, Paris, France; 3 Cardiovascular and Interventional Radiology Department, AP-HP, Pitié-Salpêtrière Hospital, Paris, France; 4 Vascular Surgery Department, AP-HP, Pitié-Salpêtrière Hospital, Paris, France; 5 INSERM U1088, UFR de Médecine et Pharmacie, Jules Verne University of Picardy, Amiens, France; 6 Clinical Research Centre, Division of Clinical Pharmacology, Amiens University Hospital and the Jules Verne University of Picardy, Amiens, France; 7 Division of Nephrology, Ambroise Paré Hospital, AP-HP, Boulogne-Billancourt, France; 8 Department of Endocrine and Oncologic Biochemistry, AP-HP, Pitié-Salpêtrière Hospital, Paris, France; 9 INSERM, UMR_S 1138, Centre de Recherche des Cordeliers, Paris 06, France; 10 Institute of Cardiometabolism and Nutrition ICAN, Paris, France; 11 Department of Pharmacology and CIC-1421, AP-HP, AP-HP, Pitié-Salpêtrière Hospital, Paris, France; 12 INSERM, CIC-1421, Paris, France; 13 INSERM U1018, Research Centre in Epidemiology and Population Health (CESP) Team 5, University of Paris Saclay-Versailles-St-Quentin-en-Yvelines (UVSQ), Villejuif, France; 14 INSERM U1166, Paris, France; 15 Division of Vascular Surgery, Department of Molecular Medicine and Surgery, Karolinska Institutet, Stockholm, Sweden; 16 Center for Molecular Medicine, Karolinska Institutet, Stockholm, Sweden; 17 Division of Renal Medicine, Department of Clinical Science, Intervention and Technology, Karolinska Institutet, Stockholm, Sweden

**Keywords:** Vascular calcification, Adiponectin, Type 2 diabetes, Peripheral arterial disease

## Abstract

**Background:**

Medial calcification in diabetes contributes to the arterial occlusive process occurring below the knee level. Adiponectin is an adipokine with atheroprotective properties and possible protective role against arterial calcification. The aim of the study was to investigate, in type 2 diabetes, the link between vascular expression and serum concentration of adiponectin and (1) peripheral arterial calcification and (2) lower limb occlusive arterial disease.

**Methods:**

Scoring of peripheral vascular calcification and peripheral arterial occlusive disease, using CT-scan and color-duplex ultrasonography respectively, were conducted and explored in relation to serum adiponectin level in a cross sectional study of 197 patients with type 2 diabetes. Vascular adiponectin expression in the arterial wall of diabetic patients with and without medial calcification was evaluated by immunohistochemistry.

**Results:**

Peripheral arterial calcification score was higher in patients with the highest adiponectin concentration. In a multivariate logistic regression analysis, an increase of 1 µg/mL of adiponectin was associated with a 22% increase of arterial calcification (adjusted OR = 1.22; 95% CI 1.03–1.44; p = 0.02). Arterial occlusive score was also higher in patients with adiponectin concentration > median (2.8 ± 4.8 vs 4.2 ± 5.7, p = 0.034). Immunohistochemical analyses showed a strong and specific staining of adiponectin in smooth muscle cells in calcified arteries, with a more pronounced expression of adiponectin in early stages of medial calcification.

**Conclusions:**

Peripheral arterial calcification is positively associated with circulating adiponectin levels in patients with type 2 diabetes, but vascular adiponectin expression is already observed at early stages of calcification. Adiponectin secretion could be a compensatory mechanism against the calcification process.

*Trial registration* DIACART NCT number: NCT02431234. Registered 30 April 2015

## Background

Peripheral arterial occlusive disease (PAOD) is a severe complication of diabetes because of the associated risk of lower limb amputation [1]. This risk is particularly high when calcification of the arterial wall coexists with PAOD [2]. Thus, preventing arterial wall calcification is a therapeutic challenge, but it requires a better understanding of the mechanisms involved. We previously showed that there is no link between arterial calcification at the below knee level and several inflammatory markers or OPG/RANKL (Osteoprotegerin/Receptor Activator of Nuclear Factor Kappa-B ligand) serum levels in patients with type 2 diabetes (T2D) and a high cardiovascular risk [3].

Among other factors potentially implicated in arterial wall calcification, adiponectin could play a protective role. Indeed, Luo et al. have shown that adiponectin, in addition to its anti-atherogenic and protective endothelial function properties, reduces arterial calcification [4, 5], possibly by inhibiting apoptosis and endoplasmic reticulum stress in vascular smooth muscle cells (VSMC) [6]. Furthermore in T2D a low level of total adiponectin co-exists with a high rate of cardiovascular events [7]. Because patients with T2D are more likely to develop arterial calcification, we hypothesized that low levels of adiponectin could be associated with the development of arterial calcification in this population, particularly in lower limbs, which is a characteristic of diabetes.

To test this hypothesis we accurately scored lower limb vascular calcification and investigated, in a cross-sectional study, whether there was a relationship between serum adiponectin levels and (1) lower limb arterial calcification and (2) peripheral arterial occlusive disease. Because systemic adiponectin concentration might not reflect the local adiponectin expression, we also explored the vascular adiponectin expression in human calcified arteries.

## Materials and methods

### Study design

This cross-sectional study (the DIACART study, for Diabète et Calcification Arterielle) was performed over an 8-month period, during which 197 people with T2D from the Diabetes and Cardiology Departments of the Pitié-Salpêtrière Hospital (Paris, France) were included [3]. Inclusion criteria were type 2 diabetes with at least 1 of the following risk factors: coronary artery disease (CAD) or peripheral arterial occlusive disease or age > 50 years for men and > 60 years for women. Exclusion criteria were (1) an estimated glomerular filtration rate (eGFR) < 30 mL/min by the modification of diet in renal disease equation (MDRD), (2) a history of lower limb angioplasty and/or bypass. The study was approved by the local ethics committee and registered in ClinicalTrials.gov (Identifier: NCT02431234). All patients were informed on the study objectives and procedure. Participants gave their written informed consent to participation.

We dispose also of artery samples (inferior epigastric artery) obtained from five end-stage renal disease patients with T2D, undergoing living donor kidney transplantation at Karolinska University Hospital, Stockholm Sweden. All patients provided written informed consent and the study protocol was approved by the Regional Ethical Review Board in Stockholm.

### Study protocol

All patients had clinical evaluation, laboratory blood tests, color duplex ultrasonography, and a multislice spiral CT scan. Patient interviews focused on comorbidities and personal disease history. Patients’ medical records were reviewed to check the information given and to record concomitant treatments. Previous cardiovascular disease was defined as a history of any of myocardial infarction, stroke, or any surgical procedures undergone for CAD. Peripheral neuropathy was evaluated by the neuropathy disability score (NDS) with NDS ≥ 6 being considered as abnormal [8].

### Laboratory evaluations

Blood and urine samples were collected after an overnight fast for the measurement of routine biochemistry tests, high-sensitivity C-reactive protein (hsCRP), interleukine 6 (IL-6), calcium, phosphate, 25-hydroxyvitamin D, and intact parathyroid hormone (iPTH). Circulating total adiponectin concentrations were measured in serum using an enzyme-linked immunosorbent assay kit (ALPCO, Eurobio, Paris, France) as recommended by the manufacturer. The lower detection limit was 0.4 µg/mL for total adiponectin. Inter-assay coefficients of variation of low and high human pool controls were 7.93% and 8.46% for total adiponectin.

### Assessment of peripheral arterial occlusive disease, occlusive score, and mediacalcosis score by color duplex ultrasonography

Detailed color duplex ultrasonography was conducted by physicians from the abdominal tree down to the foot arteries. Occlusive disease was defined as the presence of either > 70% stenosis or an occlusion in aorto-ilio-femoral segment, popliteal artery, tibio-peroneal trunk, anterior tibial, posterior tibial, peroneal, or dorsalis pedis arteries. The results were scored according to an angiographic score based on the severity of the stenosis in the lower limb arteries: 0 if stenosis was < 70%, 2 if stenosis was > 70%, and 3 in case of occlusion; duplex scores ranged from 0 to 39 [3].

### Imaging for below-knee arterial calcification score by CT scan

Below-knee artery calcification scores were obtained after scanning with a 128-slice multidetector CT scanner (Somatom Definition Flash; Siemens Healthcare) without contrast, in a craniocaudal direction, from the bottom of the patella down to the ankle region. The 3-mm cross-sectional slices were separately analyzed. Analysis was performed by one radiologist unaware of the results of the color duplex ultrasonography, laboratory tests, and clinical examinations, using a commercially available software package (Heartbeat CaScore; Philips Healthcare).

On cross-sectional images, areas of calcification along below-knee arteries with a density ≥ 130 Hounsfield units attenuation and a surface > 1 mm^2^ were identified automatically. Calcification score, determined according to the method described by Agatston et al. [9], was obtained separately for each of the main below-knee arteries (distal popliteal, anterior tibial, posterior tibial, and peroneal arteries) and added up to obtain the calcification score [3].

### Immunohistochemical staining of adiponectin in diabetic arteries

Artery samples were immediately fixed in 4% phosphate buffered formalin and embedded in paraffin. Tissue sections (1–2 µm thick) were stained with hematoxylin and eosin and van Kossa, respectively, and were evaluated by an experienced pathologist. The degree of medial calcification was semi-quantified on the van Kossa stained sections and graded 0–3: 0 = no media calcification; 1 = mild media calcification; 2 = moderate media calcification and 3 = severe media calcification as previously described [10].

In immunohistochemical staining, isotype rabbit IgG was used as a negative control while adiponectin was detected using rabbit anti-adiponectin antibody (#PA1-84881, Invitrogen). All reagents were from Histolab (Sweden). In brief, paraffin sections were deparaffinized in Tissue Clear and rehydrated in graded ethanol. For antigen retrieval, slides were subjected to high-pressure boiling in DIVA buffer (pH 6.0). After blocking with Background Sniper, primary antibody was diluted in Da Vinci Green solution, applied on slides and incubated at room temperature for 1 h. A probe-polymer containing alkaline phosphatase was applied, with subsequent detection using Warp Red chromogen. Slides were counterstained with Hematoxylin QS (Vector Laboratories, Burlingame, CA), dehydrated and mounted in Pertex (Histolab, Gothenburg, Sweden). Images were taken using an automated ScanScope slidescanner, magnifications are indicated in images with size bars.

### Statistical analysis

Data are expressed as either the mean ± SD, the median (for variables with a non-Gaussian distribution) or the frequency, as appropriate. Patients were stratified according to the median adiponectin level. Intergroup comparisons were made using a χ^2^ test for categorical variables and Student’s t test or the Kruskall–Wallis test for continuous variables. For variables with a non-Gaussian distribution, log-normalized values were considered in tests that assumed normally distributed variables. Univariate logistic regression analyses were performed to evaluate the association between adiponectin levels (categorized by the median) and selected demographic, biochemical and clinical variables. Thereafter, a multiple logistic regression analysis of the factors selected in the univariate analysis was carried out to identify those independently associated with adiponectin levels (Table 2). Univariate logistic regression analyses were performed to evaluate the association between calcification score (categorized by the median)/occlusive arteriopathy (absence vs presence) and selected demographic, biochemical and clinical variables. Thereafter, multiple logistic regressions analysis of the factors selected in the univariate analysis were carried out to identify those independently associated with calcification score (Table 3) and occlusive arteriopathy (Table 4). A P value ≤ 0.05 was considered to be statistically significant. All statistical analyses were performed using SPSS software (SPSS Inc, Chicago, IL), version 13.0 for Windows (Microsoft Corp, Redmond, WA).

## Results

### Baseline characteristics

Table 1 shows the main clinical and biochemical characteristics for the entire cohort (197 subjects) and as a function of the median of adiponectin levels (3.5 µg/mL). Eighty percent of the patients were men, with a mean age of 64 ± 8 years, diabetes duration of 15 ± 10 years, and body mass index (BMI) of 29 ± 5 kg/m^2^. Sixty percent of the subjects were active or former smokers, 82% had hypertension, 89% were treated with statins, 76% had CAD and 43% had PAOD. Mean hemoglobin A1c (HbA1c) was 7.8% ± 1.5% (62 ± 9.3 mmol/mol), and 47% of the patients were on insulin treatment. Mean eGFR was 76 ± 20 mL/min. Mean ± SD and median calcification scores were 2528 ± 5779 and 524, respectively. In 110 patients (56%), calcification scores were higher than 400, a score considered as severe [2].

**Table 1:** Baseline characteristics as a function of the median of adiponectin levels

	All n = 197	Adiponectin < 3.5 µg/mL n = 98	Adiponectin ≥ 3.5 µg/mL n = 99	P
Adiponectin (µg/mL)	4.1 ± 2.6 (3.5)	2.5 ± 0.6 (2.6)	5.7 ± 2.8 (5.0)	NA
*Age (years)*	*64 ± 8*	*62 ± 9*	*67 ± 8*	*< 0.0001*
Diabetes duration (years)	15 ± 10	14 ± 9	15 ± 9	0.322
*Male gender n (%)*	*157 (80)*	*86 (88)*	*71 (72)*	*0.007*
Body mass index (kg/m^2^)	29 ± 5	29 ±*5*	29 ± *6*	0.441
SBP (mmHg)	127 ± 17	125 ± 17	129 ± 17	0.123
DBP (mmHg)	73 ± 9	73 ± 9	73 ± 9	0.472
*Smoking habit n (%)*	*118 (60)*	*69 (70)*	*49 (50)*	*0.004*
Previous CVD n (%)	138 (70)	71 (72)	67 (68)	0.534
Glycaemia (mmol/L)	8.2 ± 2.8 (7.8)	8.3 ± 2.8 (7.8)	8.0 ± 2.8 (7.4)	0.391
HbA1c (%)	7.8 ± 1.5 (7.5)	7.8 ± 1.4 (7.7)	7.7 ± 1.5 (7.4)	0.408
*GFR MDRD (mmol/L)*	*76 ± 20*	*80 ± 19*	*72 ± 20*	*0.003*
Microalbuminuria (mg/L)	166 ± 842 (23)	100 ± 322 (20)	230 ± 1144 (23)	0.328
Calcium (mmol/L)	2.30 ± 0.11	2.32 ± 0.09	2.32 ± 0.13	0.885
Phosphate (mmol/L)	1.02 ± 0.15	1.01 ± 0.15	1.04 ± 0.16	0.165
*Intact PTH (pg/mL)*	*54.7 ± 27.5 (46.9)*	*48.8 ± 24.7 (42.9*	*60.6 ± 29.0 (54.0)*	*0.001*
25(OH)Vit D (ng/mL)	13.8 ± 8.4 (12.0)	14.0 ± 8.7 (12.0)	13.7 ± 8.1 (12.0)	0.924
*hsCRP (mg/L)*	*2.2 ± 1.5 (1.2)*	*2.5 ± 2.6 (1.5)*	*1.9 ± 2.3 (0.8)*	*0.035*
*IL6 (pg/mL)*	*5 ± 22 (3)*	*7 ± 31 (3)*	*3.3 ± 3.9 (2.7)*	*0.045*
*Triglycerides (mmol/L)*	*1.6 ± 1.1 (1.3)*	*1.8 ± 1.2 (1.4)*	*1.4 ± 0.8 (1.1)*	*0.001*
Total cholesterol (mmol/L)	3.7 ± 0.9	3.6 ± 0.9	3.8 ± 0.9	0.146
LDL cholesterol (mmol/L)	1.9 ± 0.7 (1.8)	1.9 ± 0.7 (1.8)	2.0 ± 0.8 (1.8)	0.097
*Total cholesterol/HDL cholesterol*	*3.7 ± 1.6 (3.5)*	*4.1 ± 1.9 (3.7)*	*3.4 ± 1.0 (3.3)*	*< 0.0001*
*NDS*	*2.4 ± 2.4 (2.4)*	*2.1 ± 2.4 (2)*	*2.7 ± 2.4 (2)*	*0.035*
*Peripheral calcification score*	*2528 ± 5779 (524)*	*1818 ± 5283 (290)*	*3239 ± 6204 (1044)*	*0.004*
*Occlusive score*	*3.5 ± 5.3*	*2.8 ± 4.8*	*4.2 ± 5.7*	*0.034*
Insulin use n (%)	93 (47)	45 (46)	48 (49)	0.414
*Metformin use n (%)*	*160 (81)*	*88 (90)*	*72 (73)*	*0.002*
Statin use n (%)	175 (89)	88 (90)	87 (88)	0.421
ARB and ACE inhibitors use n (%)	158 (80)	81 (83)	77 (78)	0.249

Data are given as mean ± SD for normally distributed measures with addition of (median) for non-normally distributed values for variables with a non-Gaussian distribution or as the number (percentage) for binary variables; variables mentioned in italic are variables distributed significantly different in both groups

*NA* not applicable, *ARB* angiotensin receptor blockers, *ACE* angiotensin converting enzyme, *SBP* systolic blood pressure, *DBP* diastolic blood pressure, *CVD* cardiovascular disease, *HbA1c* haemoglobin A1C, *GFR MDRD* glomerular filtration rate calculated with the modification of diet in renal disease formula, *PTH* parathyroid hormone, *hsCRP* high sensibility C-reactive protein, *IL-6* interleukin 6, *NDS* neuropathy disability score

### Characteristics of the population according to total adiponectin levels

Mean and median total adiponectin levels were 4.1 ± 2.6 and 3.5 µg/mL, respectively (Table 1). Patients with serum adiponectin concentration higher than the median, were older, more frequently women and less often smokers. They had a lower GFR, IL-6, total-cholesterol/HDL ratio and triglycerides levels, but higher iPTH levels. After multivariate logistic regression analysis, only age (per one year, OR = 1.06; 95% CI 1.02–1.11; P 0.001), male gender (OR = 0.388; 95% CI 0.173–0.872; P 0.022), iPTH concentration (OR = 1.013; 95% CI 1.000–1.03; P 0.032), and total cholesterol/HDL-cholesterol ratios (OR = 0.645; 95% CI 0.486–0.856; P = 0.002) were significantly associated with the adiponectin level (Table 2).

**Table 2:** Multivariate logistic regression analysis: variables independently associated with the Adiponectin split by the median (n = 197 patients)

	Odds ratio (95% confidence interval)	P
Age per 1 year	1.06 (1.02; 1.11)	0.001
PTH per 1 pg/mL	1.013 (1.00; 1.03)	0.032
Male gender	0.388 (0.173; 0.872)	0.022
Total cholesterol/HDL cholesterol	0.645 (0.486; 0.856)	0.002

Variables entered into the model: age, gender, PTH, Triglycerides, Total cholesterol/HDL cholesterol, GFR MDRD

For abbreviations, please refer to Table 1

### Variables associated with peripheral arterial calcification

**Fig. 1:**
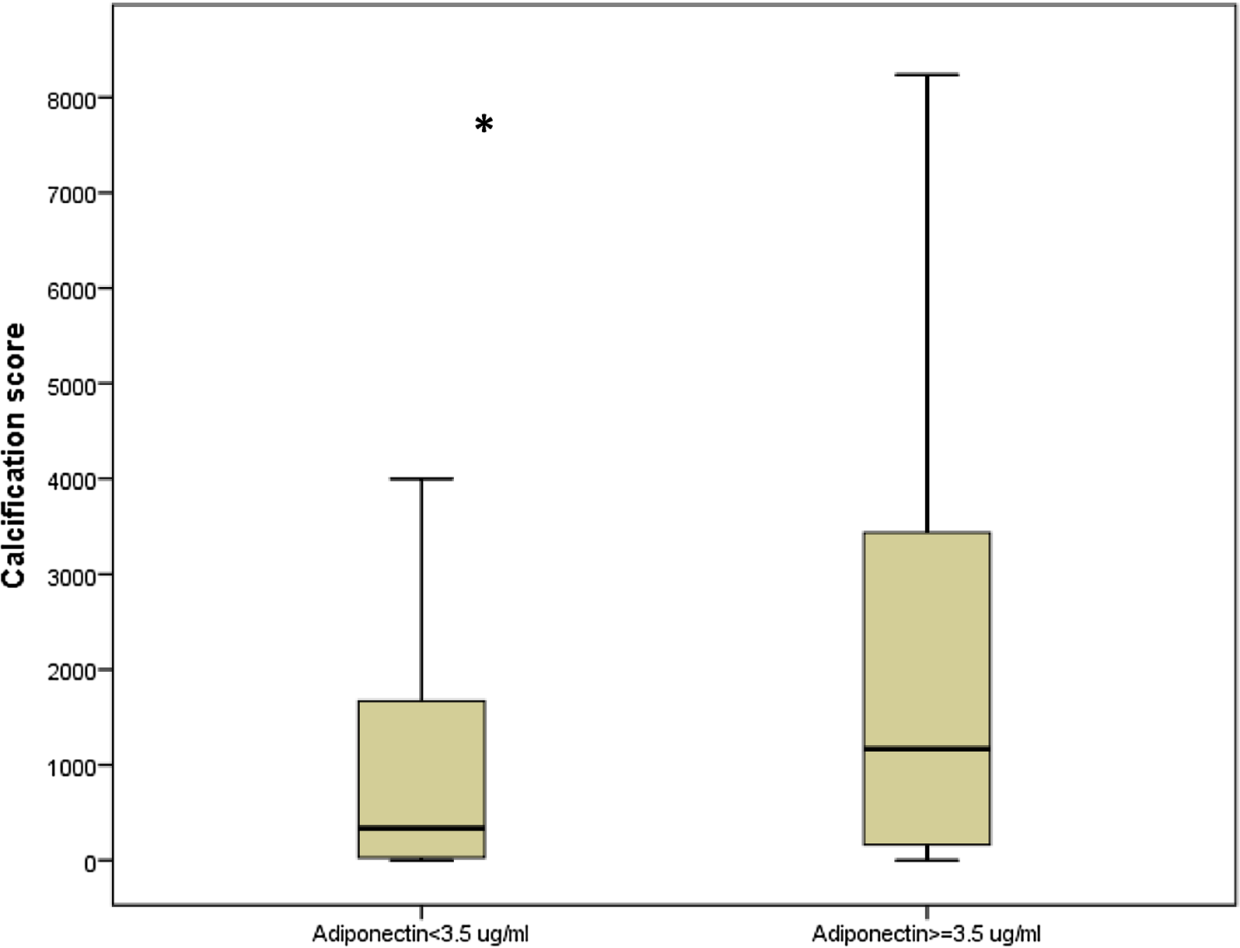
Calcification score according to adiponectin levels (*corresponding P = 0.004). Boxplot distribution of participant age for each donning instruction condition with the lower extreme, the lower quartile, median, upper quartile and upper extreme. Lower limb calcification score is expresses in both groups of patients lower or higher to the median of adiponectin levels (3.5 µg/mL). Data are given as mean ± SD for normally distributed measures with addition of (median) for non-normally distributed values for variables with a non-Gaussian distribution or as the number (percentage) for binary variables. *NA* not applicable, ARB

In univariate logistic regression analysis, age, adiponectin, gender, previous cardiovascular disease, and neuropathy were associated with peripheral calcification score, whereas diabetes duration, smoking status, BMI, systolic and diastolic blood pressure, GFR-MDRD, glycaemia, HbA1c, microalbuminuria, Il-6, hsCRP, cholesterol, 25(OH)vitamin-D, calcium, phosphate, and metformin use were not. In multivariate logistic regression analysis, calcification score was correlated with age (per one year, OR = 1.07; 95% CI 0.486–0.856; P = 0.002), male gender (OR = 4.17; 95% CI 1.75–9.95; P = 0.001), previous cardiovascular disease (OR = 2.71; 95% CI 1.33–5.50; P = 0.012), NDS (per one point, OR = 1.19; 95% CI 1.04–1.37; P = 0.010) and total adiponectin (per 1 µg/mL, OR = 1.22; 95% CI 1.03–1.44; P = 0.020) (Table 3). Figure 1 presents calcification score according to adiponectin levels.

**Table 3:** Multivariate logistic regression analysis: variables independently associated with the calcification score split by the median (n = 197 patients)

	Odds ratio (95% confidence interval)	P
Age per 1 year	1.07 (1.02; 1.11)	0.003
Adiponectin per 1 µg/mL	1.22 (1.03; 1.44)	0.024
Male gender	4.17 (1.75; 9.95)	0.001
Previous CVD	2.71 (1.33; 5.50)	0.006
NDS per 1 point	1.19 (1.04; 1.37)	0.010

Variables entered into the model: age, adiponectin, gender, previous CVD, NDS

For abbreviations, please refer to Table 1

### Variables associated with lower limb arterial occlusive disease

**Fig. 2:**
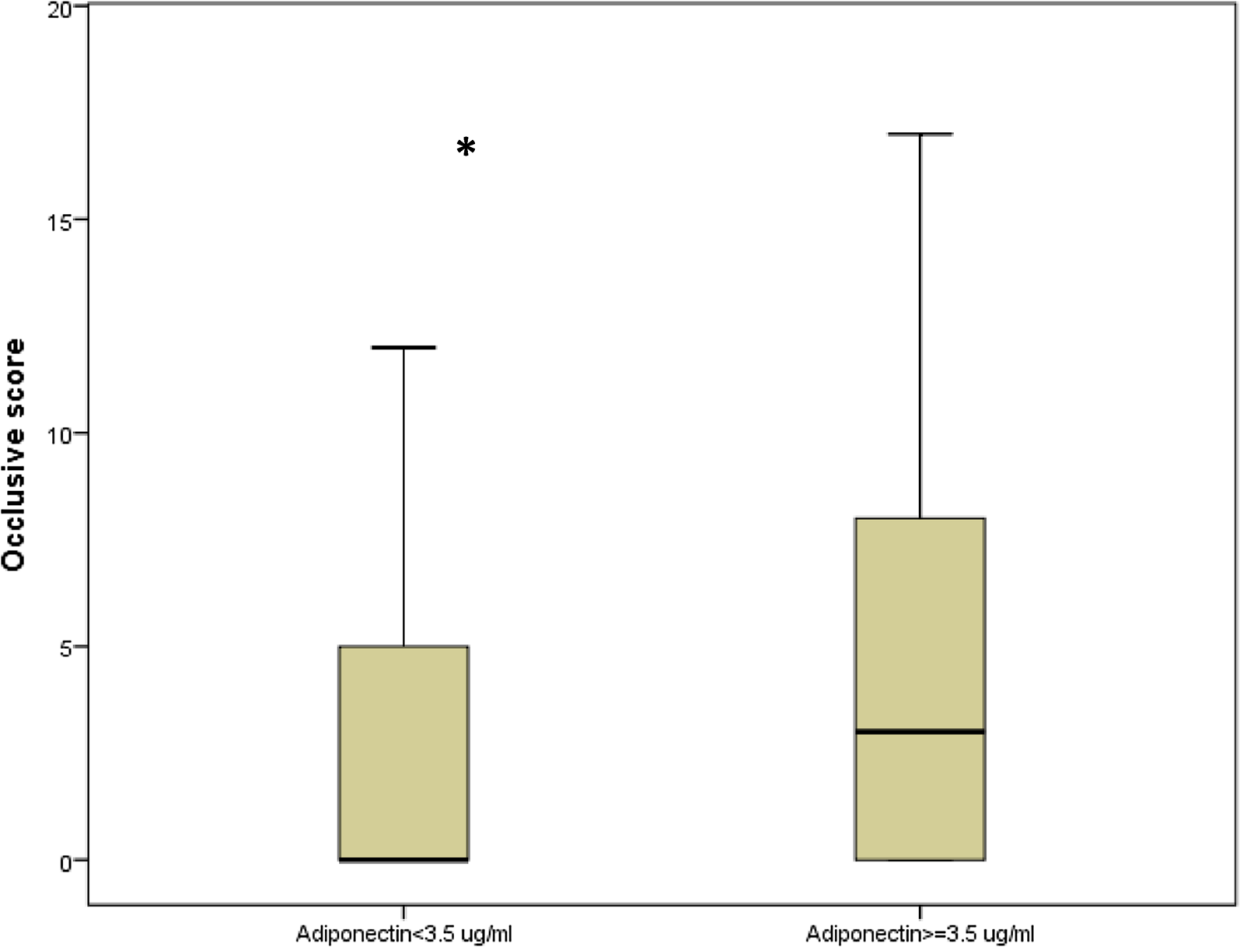
Occlusive score according to adiponectin levels (*corresponding P = 0.034). Boxplot distribution of occlusive score as a function of adiponectin concentration lower or higher than the median of adiponectin concentration in the population. Data are given as the lower extreme, the lower quartile, median, upper quartile and upper extreme

In univariate logistic regression analysis, age, diabetes duration, adiponectin, previous cardiovascular disease, systolic blood pressure, iPTH, total cholesterol/HDL ratio and NDS were associated to the presence of occlusive arteriopathy. Gender, smoking status, BMI, diastolic blood pressure, GFR-MDRD, glycaemia, HbA1c, microalbuminuria, IL-6, cholesterol, CRP, 25(OH)-vitamin-D, calcium, and phosphate were not. In multivariate logistic regression analysis, presence of occlusive arteriopathy was independently associated with systolic blood pressure (per 1 mmHg, OR = 1.02; 95% CI 1.00–1.04; P = 0.015), iPTH (per 1 pg/mL, OR = 1.02; 95% CI 1.00–1.03; P = 0.022), previous cardiovascular disease (OR = 2.18; 95% CI 1.08–4.43; P = 0.006), NDS (per one point, OR = 1.25; 95% CI 1.09–1.43; P = 0.031) and total adiponectin (per 1 µg/mL, OR = 1.16; 95% CI 1.00–1.33; P = 0.022) (Table 4). Figure 2 shows the occlusive score in patients with adiponectin levels below and ≥ median.

**Table 4:** Multivariate logistic regression analysis: variables independently associated with occlusive arteriopathy (absence vs presence)

	Odds ratio (95% confidence interval)	P
SBP per 1 mmHg	1.02 (1.00; 1.04)	0.015
Adiponectin per 1 µg/mL	1.16 (1.00; 1.33)	0.044
PTH per 1 pg/mL	1.02 (1.00; 1.03)	0.022
Previous CVD	2.18 (1.08; 4.43)	0.006
NDS per 1 point	1.25 (1.09; 1.43)	0.031

Variables entered into the model: Age, diabetes duration, Previous CVD, SBP, NDS, PTH, adiponectin, cholesterol total/HDL (not selected in the final model: age, diabetes duration, cholesterol total/HDL)

For abbreviations, please refer to Table 1

### Adiponectin expression in vascular wall of patients with type 2 diabetes

**Fig. 3:**
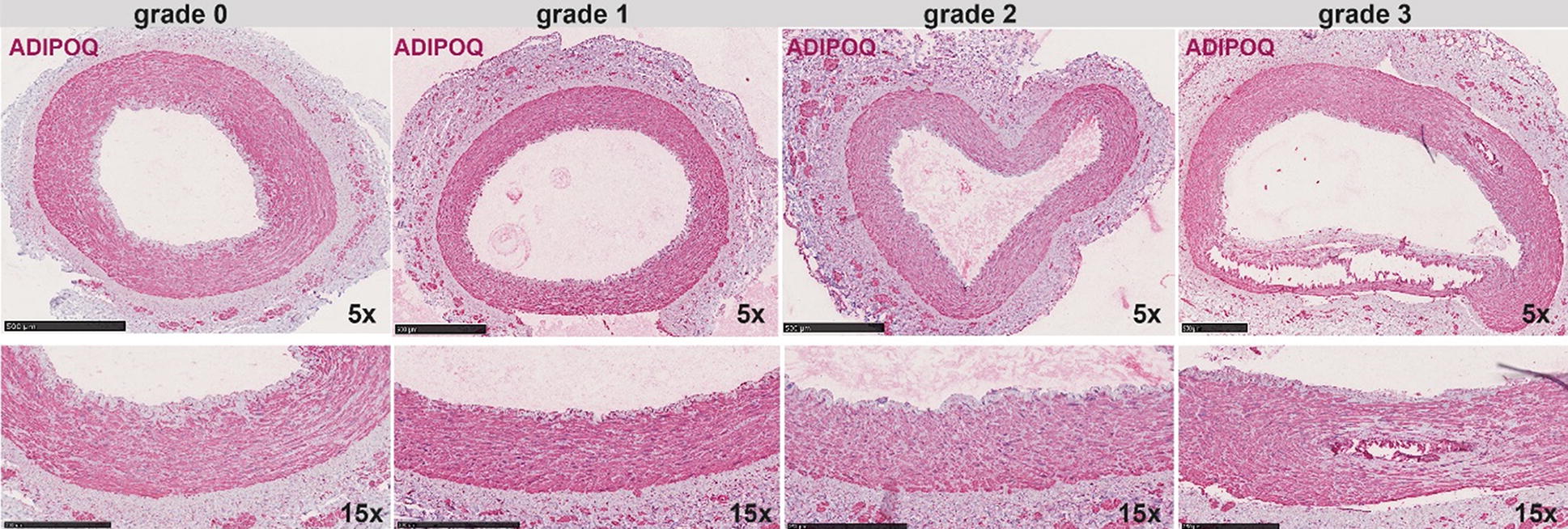
Immunohistochemical staining of adiponectin in arteries from patients with type 2 diabetes. Adiponectin (ADIPOQ) protein expression and localization was examined by immunohistochemistry staining in arteries from diabetic patients, classified according to the grade of calcification. ADIPOQ (red signal) was found to be expressed in all stages of calcification, but it was particularly enriched in early stages (grade 1). Smooth muscle cells of the media were specifically immuno positive for ADIPOQ, but the signal was also present in microvessels of the adventitia (particularly grade 2). Images were taken with ×5 magnification and enlarged images with ×15 (size bars included)

A pronounced expression of adiponectin by immunohistochemistry was observed in calcified arteries from diabetic patients (Fig. 3). Adiponectin was particularly strongly expressed in early stages of calcification (grade 1) compared to other stages, and specifically localized in medial smooth muscle cells. In arteries classified with grade 2 of calcification, the muscular staining for adiponectin appeared to be more intense in the adventitia. The presence of major calcifications (grade 3) did not associate with stronger staining for adiponectin in comparison with uncalcified diabetic arteries.

## Discussion

We describe for the first time, in a well-characterized population of patients with type 2 diabetes with high cardiovascular risk but without severe kidney disease, a positive association between total adiponectin levels and peripheral arterial calcification severity, even after adjustment for multiple confounders. We also show in this population that total adiponectin concentration is associated with the severity of peripheral arterial occlusive disease. Furthermore, in situ, we show strong adiponectin expression in muscular layer of calcified arteries. We also report that the arterial adiponectin expression is particularly enriched in the early stage of media calcification and not so much in the late stage of extended calcification (i.e., grade 3). These results suggest that arterial adiponectin expression and serum adiponectin concentrations do not change in parallel and circulating adiponectin does not reflect the arterial adiponectin expression.

A lot of factors are known to be associated with lower limb arterial calcification (age, sex, previous cardiovascular disease, eGFR, NDS, PTH, inflammation and metformin) [3, 11–13]. It is interesting to note that there is well described relationship between adiponectin and most of these parameters (age, sex, cardiovascular disease, kidney function, lipids, bone remodeling, inflammation, neuropathy and metformin) [14–17]. In our study, adiponectin concentration was independently associated after multivariate analysis with age, male sex, total cholesterol/HDL cholesterol and iPTH. Finally, only age, sex, previous cardiovascular disease, NDS and adiponectin were associated independently after multivariate analysis with below the knee arterial calcification.

The positive association between serum adiponectin and peripheral arterial calcification observed in the current cohort is surprising because some experimental data suggest that adiponectin has an inhibitory function against arterial calcification. Indeed, in vitro, VSMC express type 1 adiponectin receptor (AdipoR1), and treatment by adiponectin reduces osteoblastic differentiation of VSMC through the AdipoR1/p38 mitogen-activated protein kinase (MAPK) signaling pathway [5]. Another pathway for adiponectin inhibitory effect on VSMC osteoblastic differentiation could be the regulation of AMP activated protein Kinase/mTOR activity [18]. Adiponectin could also reduce vascular calcification development by inhibiting apoptosis and endoplasmic reticulum stress in VSMC [6]. In vivo, adiponectin deficient mice develop media calcification on the thoracic aorta, which is attenuated by treatment with adiponectin adenovirus [5]. Furthermore, in healthy people and in type 1 diabetic patients with a low cardiovascular risk, low plasma adiponectin levels were independently associated with progression of coronary artery calcification (CAC) [19]. Thus, globally adiponectin seems to have anti-calcifying properties. Thus, in our cohort high circulating adiponectin concentration could be a marker rather than a causal factor for peripheral arterial calcification. Another argument for this hypothesis is that we did not observe a positive correlation between arterial adiponectin protein levels in situ and arterial calcification gradation. The results are in favor of a local vascular protective counter regulation process, which may explain why the increase of arterial adiponectin expression is only observed during initiation of vascular calcification (stage 1). As adiponectin has in vitro calcification inhibitory properties, increased local adiponectin expression could be a compensatory mechanism against nascent osteoblast-like cells observed in arterial wall during the first stages of calcification process itself, following a feedback loop. Indeed adiponectin secretion is stimulated in presence of osteoblasts and calcification [20].

The increase of serum adiponectin concentration, that is associated with arterial calcification, should not be considered as a causal factor of calcification but may rather be a consequence of arterial calcification. Indeed arterial calcification induces a stiffening of the arterial wall, which may result in decreased vascular compliance, increased pulse wave velocity and pulse pressure, increased cardiac afterload and a subsequent left ventricular hypertrophy and heart failure. Interestingly, in patients with heart failure, high serum adiponectin concentrations and severity of the disease are correlated [21–26]. These associations could be explained by muscular adiponectin resistance. Indeed, Van Berendoncks et al. have described a fivefold increase in the adiponectin expression with downregulation of adiponectin receptor in skeletal muscle cells of patients with mild to moderate chronic heart failure [27]. In our patients it is possible that arterial calcification development led to left ventricular hypertrophy and heart failure associated with increased serum adiponectin. But this needs confirmation. Our data also accord with reports from patients with chronic kidney disease in whom high circulating adiponectin levels predict poor outcome [28]. It has been speculated that pathogenic pathways linked to the wasting process, salt overload and volume status contribute to the adiponectin serum levels increase observed in uremic clinical situation [29].

It may be hypothesized that increased circulating levels of adiponectin could be a compensatory mechanism, for example against systemic oxidative stress and inflammation which are pathophysiological pathways activated in patients with arterial calcification [30]. In patients with a high cardiovascular risk or established heart disease, systemic oxidative stress and inflammation are often observed. It is interesting to note that paradoxically in these patients, a positive association between adiponectin and cardiovascular events are constantly reported [21–26]. In our study adiponectin levels are associated with two low grade inflammation markers, IL-6 and hsCRP. Unfortunately we did not measure oxidative biomarkers.

Some confounding factors could also explain the association between high adiponectin levels and arterial calcification, but we excluded severe kidney disease and the association was independent of glomerular filtration rate. Increased production of adiponectin could be confounded by some other factors such as heart disease or drugs known to modify adiponectin concentrations such as metformin and angiotensin II blockers, but in our study the association persisted after adjustment for previous cardiovascular disease and medications.

The strengths of the current study are the objective assessment of arterial calcification by CT scan, the fine characterization of the population, the exclusion of patients with serious kidney disease, the immunohistochemistry study of calcified and uncalcified arteries of patients with diabetes. The limitations are the cross-sectional design, which does not allow for causal hypotheses, and the absence of a control population.

## Conclusion

The arterial expression and the systemic concentration of adiponectin are not associated in the same way with arterial calcification. Circulating adiponectin concentration does not reflect arterial adiponectin expression. While adiponectin does not appear to be involved in arterial calcification development, a role for adiponectin in counter regulation pathways at the early stages of arterial calcification is suggested. Further explorations are needed to confirm this hypothesis.

## Abbreviations

CAD: coronary artery disease
CAC: coronary artery calcification
CT-scan: computed tomography scan
DIACART: Study for “Diabète et Calcification Arterielle”
DSB: diastolic blood pressure
eGFR: estimated glomerular filtration rate
HbA1c: hemoglobin A1c
hsCRP: high-sensitivity C-reactive protein
IL-6: interleukine 6
iPTH: intact parathyroid hormon
MDRD: modification of diet in renal disease equation
NDS: neuropathy disability score
OPG: osteoprotegerin
PAOD: peripheral arterial occlusive disease
RANKL: Receptor Activator of Nuclear Factor Kappa-B ligand
SBP: systolic blood pressure
T2D: type 2 diabetes
VSMC: vascular smooth muscular cell
